# Age-induced NLRP3 Inflammasome Over-activation Increases Lethality of SARS-CoV-2 Pneumonia in Elderly Patients

**DOI:** 10.14336/AD.2020.0601

**Published:** 2020-07-23

**Authors:** Pedro C Lara, David Macías-Verde, Javier Burgos-Burgos

**Affiliations:** ^1^Hospital Universitario San Roque, Las Palmas, Spain; ^2^Universidad Fernando Pessoa Canarias, Las Palmas, Spain; ^3^Instituto Canario de Investigación del Cáncer, Tenerife, Spain

**Keywords:** NLRP3, inflammasome, age, COVID-19, pneumonia

## Abstract

Age is one of the most important prognostic factors associated to lethality in SARS-CoV-2 infection. In multivariate analysis, advanced age was an independent risk factor for death. Recent studies suggest a role for the nucleotide-binding domain and leucine rich repeat containing family, pyrin domain containing 3 (NLRP3) inflammasome activation in lung inflammation and fibrosis in SARS-CoV and SARS-CoV-2 infections. Increased NLRP3/ apoptosis-associated speck-like protein (ASC) mRNA expression and increased caspase-1 activity, have been observed in aged lung, provoking increased and heightened expression levels of mature Interleukin (IL)-1β and IL-18 in aged individuals. Aged individuals have a basal predisposition to over-react to infection, displaying an increased hyper-inflammatory cascade, that seems not to be fully physiologically controlled. NLRP3 inflammasome is over-activated in aged individuals, through deficient mitochondrial functioning, increased mitochondrial Reactive Oxigen Species (mtROS) and/or mitochondrial (mt)DNA, leading to a hyper-response of classically activated macrophages and subsequent increases in IL-1 β. This NLRP3 over-activated status in elderly individuals, is also observed in telomere dysfunctional mice models. In our opinion, the NLRP3 inflammasome plays a central role in the increased lethality observed in elderly patients infected by COVID-19. Strategies blocking inflammasome would deserve to be studied.

The new SARS-CoV-2 coronavirus infection is a pandemic affecting patients throughout the globe. Most symptomatic patients show mild respiratory symptoms such as dry cough, fever, and moderate dyspnea. Unfortunately, severe presentations include viral pneumonia, acute respiratory distress syndrome (ARDS) and sequential organ failure (SOF) [1].

The Coronavirus family are single-stranded RNA-viruses that can infect both animals (mainly bats) and humans. Infection leads to activation of the inflammatory T helper 1 (Th1) cells and macrophages. Activated inflammatory cells, participate in the production of several pro-inflammatory cytokines (especially IL-1β), enter the pulmonary circulation and induce the so-called “cytokine storm” that lead to rapid, wide-spread damage of the pulmonary epithelium and alveolar cells, as well as other vital organs [2].

The “cytokine storm” induced by activated lymphocytes induces a systemic disease displaying several deteriorating presentations characteristic of the critical COVID-19 illness. This hyper-inflammatory host´s response has been medically managed by interleukin inhibitors, chloroquine or steroid, that seems to effectively modulate the hyperimmune response to SARS-CoV-2 infection [3].

Poor survival for patients severely affected by SARS-CoV-2 infection has been related to pre-existing comorbidities, high serum inflammation markers (D-Dimer/Ferritin) levels, SOFA (Systemic Organ Failure Assessment) and age [1].

Infected patients with comorbidities, such as diabetes, chronic renal disease, and/or chronic pulmonary disease, are at greater risk of severe complications and mortality from COVID-19 disease. Diabetes and chronic renal disease establish a pro-inflammatory state due to defects in both innate and adaptive immunity. Furthermore, chronically damaged lung tissues, renders the pulmonary parenchyma pre-compromised, to cope with the coronavirus induced ARDS [4-6].

Elevated D-Dimers and Ferritin are well known markers of systemic hyperinflammation and predicts patient outcomes in COVID-19 disease. The elevation of these inflammatory markers is similar to those found in the Macrophage Activation Syndrome and secondary Hemophagocytic LymphoHistiocytosis (MAS/sHLH). The SOFA is also a very important prognostic factor for patients suffering from COVID-19 disease [1].

Age is one of the most important prognostic factors associated to lethality in SARS-CoV-2 infection. In the multivariate analysis, advanced age resulted an independent risk factor for dying of the disease [1]. Therefore, there is room for other factors, besides comorbidities affecting elderly people, that could explain, the extremely high proportion of deaths in this age range.

## NLRP3 Inflammasome mediates hyper-inflammation in SARS-CoV family infection

This systemic hyperinflammation in SARS-CoV-infection resembles de MAS/sHLH syndrome [3]. There is an increase in IL-1β classically activated macrophages, that produces higher levels of IL-6 and Tumor Necrosis Factor (TNF)-α.

Recent studies suggest a role for NLRP3 inflammasome activation in lung inflammation and fibrosis [7] caused by SARSCov [8] and SARS-Cov-2 infections [9].

NLRP3 is an intracellular sensor that is activated by diverse stimuli, including danger-associated molecular patterns (DAMPs) (such as silica and uric acid crystals), pathogen-associated molecular patterns (PAMPs) and multiple molecular and cellular events, including ionic flux and mitochondrial dysfunction. ROS production and lysosomal damage have been shown to trigger NLRP3 activation but, however, the specific regulatory mechanisms remain uncertain [10].

The inflammasome is a specialized cellular process that couples pathogen and stress-sensing pathways to the assembly of a signaling complex. This signaling complex is composed of the three effector subunits: the NLRP3, ASC and caspase-1 [11]. The activation of caspase-1 subsequently causes the cleavage of pro-IL-1 β, and the induction of pyroptotic cell death. IL-1β promotes the production of transforming growth factor (TGF)-β, which stimulates the collagen production by resident fibroblasts. Furthermore, IL-1β can induce the secretion of neutrophil chemokines causing neutrophil influx to the lung, promote alveolar epithelial cell death, and enhance the production of platelet-derived growth factor (PDGF). All of these mechanisms cause lung fibrosis [12].

The NLRP3 inflammasome plays a pivotal role in pulmonary inflammation related diseases as chronic obstructive pulmonary disease, asthma, and ARDS. But NLRP3 activation is also in the origin of systemic diseases, like Alzheimer’s disease, prion diseases, type 2 diabetes, and some other infectious diseases [13].

The NLRP3 inflammasome activation by the Coronaviridae family viruses in alveolar macrophages seems to be mediated through different mechanisms.

The SARS-CoV E protein, forms protein-lipid channels in ERGIC/Golgi membranes, that are permeable to calcium ions. The E protein ion-channels activity in calcium transport, boosted the activation of the NLRP3 inflammasome, leading to IL-1β overproduction. These findings strikingly link SARS-CoV E protein ionic-channels-induced ionic disturbances at the cellular level, to the immunopathological consequences and disease worsening in the infected organism [14].

Recently, the SARS-CoV unique domain (SUD), that include three macrodomains (N, M, and C), was shown to significantly up-regulate the expression of IL-1β and cytokines through NLRP3 activation, in human lung epithelial cells and in mice lung tissues [15].

The murine hepatitis virus strain-3 (MHV-3) is a member of the coronavirus family and produces fulminant hepatitis. This disease is caused by excessive inflammation in the infected liver, related to a significant elevation in IL-1β expression. The infected macrophages in mice models, showed a quick release of reactive oxygen species (ROS), suggesting a plausible viral initiation of NLRP3 inflammasome activation.

ROS production deficient mice presented reductions in NLRP3 inflammasome activation and subsequent IL-1β secretion during viral infection. Moreover, viral infected animals who were constitutively deficient in the NLRP3/Caspase-1 axis, also have reduced IL-1β induction along with ameliorated clinical signs of hepatitis. All together, these results demonstrate that the ROS/NLRP3/IL-1β axis constitutes an essential signaling pathway during coronavirus infection [16].

The SARS-CoV virus genome encodes a group of the so-called accessory Open Reading Frame proteins. The SARS ORF3a protein is one of such proteins and induce macrophage activation by several mechanisms. The ORF3a protein interacts with the Receptor Interacting Protein 3 (Rip3), which augments the oligomerization of SARS ORF3a protein, helping to drive necrotic cell death. In addition, SARS ORF3a protein, also triggers lysosomal damage and dysfunction activating the NLRP3 inflammasome. SARS 3a protein can activate caspase-1 either directly or via an enhanced potassium efflux, which triggers NLRP3 inflammasome assembly [17,18]. The ORF3a accessory protein also activates the NLRP3 inflammasome by promoting TNF receptor-associated factor 3 (TRAF3)-mediated ubiquitination of ASC. ORF3a induced pro-IL-1β transcription through activation of NF-κB, which was mediated by TRAF3-dependent ubiquitination and processing of p105. Therefore ORF3a-induced elevation of IL-1β secretion, independently of its ion channel activity, but requires NLRP3, ASC, and TRAF3 activation. [19]

Another ORF protein (ORF8b) induces endoplasmic reticulum (ER) stress, lysosomal damage, and subsequent activation of Transcription factor EB (TFEB) regulating autophagy and lysosome machinery. The ORF8b protein robustly activates the NLRP3 inflammasome in macrophages. Mechanistically, ORF8b interacts directly with the Leucine Rich Repeat domain of NLRP3 and localizes with NLRP3 and ASC in cytosolic dot-like structures. ORF8b triggers cell death consistent with pyroptotic cell death in macrophages [8].

Most of SARS-CoV proteins are highly conserved (95%-100%) in SARS-CoV-2 virus protein repertoire. The SARS-CoV-2-ORF8 protein displays only a 30% identity to the SARS-CoV ancestor [20]. The ORF8 gene show a wide range of evolutionary mutations and its impact in the increased lethality of this novel infection must be addressed [21].

## NLRP3 Inflammasome mediates a pro-inflammatory status in aged individuals

Aging is associated to increased lethality in countries suffering from this pandemic. Older age has also been reported as an important independent predictor of mortality in SARS and MERS infections [22,23].

Elderly patients suffering of SARS-CoV, MERS, and SARS-CoV-2 pneumonia, develop a pulmonary disease, resembling in some aspects of pulmonary fibrosis. Interstitial lung diseases of fibrotic origin is common in patients older than 50 years [24]. Several studies have suggested, that activated alveolar macrophages and their mediators, may play a role in the pathogenesis of pulmonary fibrosis [25].

As stated above, there is room for the hypothesis, that immune response to an external insult (ie. SARS-CoV-2) would be intrinsically different, from young and elderly individuals. NLRP3 inflammasome plays a pivotal role in pulmonary inflammation related diseases (chronic obstructive pulmonary disease, asthma, and ARDS) [12]. Whether the different basal activation of NLRP3 inflammasome in young or elderly individuals, would be in the origin of such clinical differences deserves to be analyzed.

In an animal model, young and aged C57BL/6 mice, were treated with identical doses of bleomycin. Bleomycin generates intracellular ROS, which can injure lung epithelial cells and activate the NLRP3 inflammasome in macrophages, leading to IL-1β secretion. After administration of bleomycin by oral aspiration, an increased inflammatory response, lung collagen deposition and death in aged mice, was observed, compared to younger ones [26].

These pathological changes, observed in the lungs of older mice, were correlated to changes in the inflammasome-activation axis. Increased NLRP3/ASC mRNA expression /assembly and caspase-1 activity were observed in aged lungs compared to those of younger individuals.

As expected, this increased activity of the NLRP/ASC/Caspase-1 axis, resulted in increased and heightened expression levels of mature IL-1β and IL-18 in aged lungs. Therefore, an external damage signal (bleomycin) induced higher cytokine production by the aged macrophages, through NLRP3 inflammasome activation [26].

The mechanism involved in this age-related propensity to suffer inflammatory related diseases, has been addressed, highlighting the role of the mitochondrial activity. Aging is associated to a deterioration of mitochondrial performance, displaying diminished mitochondrial metabolism, increased oxidative stress, and higher levels of mtROS production. An increase in mitochondrial DNA (mtDNA) mutations, has also been observed [27]. Therefore, aging is associated to increased levels of mtROS [28], and consequent accumulation of damaged mitochondria, that could result in the release of mtDNA, that acts as DAMPs to initiate cellular responses [29]. These signals are able to spark the formation of the NLRP3 inflammasome [30].

Mitochondrial dysfunction remains as an active player of the intrinsic hyper-inflammatory background in older individuals. The immunological constitutive conditions (mitochondrial failure) of older individuals facing an external oxidative stress stimulus, can potentiate hyper-inflammatory syndromes and lung damage [31].

Therefore, aged macrophages deal with higher levels of ROS, that resulted in increased mitochondrial failure and increased activation of mitochondria-dependent apoptotic pathways [32]. These changes are detected by the NLRP3 inflammasome, induced increased assembly with the ASC protein and increased caspase-1 activation, resulting in enhanced and maintained secretion of IL-1β and IL-18. Heightened activation of the NLRP3 inflammasome, can result in increased acute lung injury [33].

Telomere shortening is a major landmark of aging. In aged individuals, telomeres gradually shorten with the continuous division of somatic cells and may reach a dysfunctional state, causing impaired host response to cellular stress, inflammatory responses [34] and mitochondrial defective function [35].

Telomere dysfunction activates p53-mediated cellular responses including repression of peroxisome proliferator-activated receptor gamma, coactivator 1 alpha and beta (PGC-1α and PGC-1β). PGCs are major regulators of mitochondrial physiology and metabolism. Therefore, telomere dysfunction is associated with impaired mitochondrial biogenesis and function, decreased gluconeogenesis, cardiomyopathy, and increased reactive oxygen species. This telomere-p53-PGC axis contributes to organ and metabolic failure and to diminishing organic fitness in the setting of telomere dysfunction [36]. Telomerase defective mice showed macrophage´s mitochondrial abnormalities, oxidative stress, and hyperactivation of the NLRP3 inflammasome [36].

As stated above, a mtROS excess is a common cause of the pro-inflammatory status, observed in aged individuals. In fact, increased levels of ROS, were the messengers that signals telomere defects to inflammasome´s activation [37].

Host´s ability to successfully cope with infection, is related to the capability of eradicating the pathogens, but also, to the ability of physiologically end up the inflammatory response, reducing the tissue damage [38]. Telomere-deficient macrophages in mice, have normal baseline inflammasome signaling and do not express any immunological disorder. But these telomerase-defective mice, displayed exaggerated lung inflammation and increased mortality, upon respiratory staphylococcal infection, although their pathogen-clearing capacity was uncompromised. Therefore, the telomere dysfunction in lung macrophages, would be a baseline over-reacting factor. It is proposed that the additive ‘‘hits’’ of a viral infection, would be required to show an abnormal immunological response, that cause unexpected clinical manifestations [36].

Therefore, the overactivation of NLRP3 inflammasome in aged individuals is caused by telomere dysfunction that, through the telomere-p53-PGC axis, contribute to the already existing mitochondrial failure, accumulating ROS and mtDNA. These higher levels of ROS and mtDNA lead to continuous activation of NLRP3 inflammasome and elevated levels of pro-inflammatory cytokines. These mechanisms further extend the concept of Inflammaging, defined as the systemic phenotype of increased basal levels of circulating cytokines in old age.

## NLRP3 mediates hyperinflammation in aged individuals infected by Coronaviruses

As described above, older age is an independent prognostic factor for survival in individuals infected by Coronaviruses. Aged individuals could display an exaggerated immune response to infections.

Recent evidence shows that lung macrophages in older animals, are in a pro-inflammatory state and are more readily activated upon infection, compared to the resting resident macrophages in young mice lungs [39]. These aged-lung resident magrophages produce increased basal levels of the pro-inflammatory cytokines IL-1β, IL-6 and TNF-α. Furthermore, the immune system in aged individuals, is in a hyper-responsive state, which can result in ARDS during severe avian influenza viral infection [40].

This preexisting pro-inflammatory status in older individuals, that resulted in an increased inflammation response to external insults, was confirmed in older macaques infected with SARS-CoV. SARS-CoV-infected aged macaques develop more severe pathology and increased lethality than younger animals, even though viral replication levels are similar.

Gene expression analysis in SARS-CoV-infected macaques revealed that the host response to SARS-CoV infection is similar in nature but differs significantly in severity in pro-inflammatory responses in aged individuals. Aged macaques had a stronger host response to virus infection than young adult macaques, with an increase in differential expression of genes associated with inflammation related to the NF-κB pathway [41].

These observations are explained by the age-related accumulated oxidative damage. This mitochondrial failure causes a disturbance in the redox balance, resulting in increased ROS. Subsequently activating the redox-sensitive transcription factors, such as NF-κB, followed by the activation of the NLRP3 inflammasome and induction of pro-inflammatory cytokines like IL-1β, IL-6 and TNFα.[42]A recent study in a small group of macaques infected with SARS-CoV-2, replicated the previous results, demonstrating an increased rate of severe interstitial pneumonia in older individuals, compared to younger ones [43].

In humans, the age-dependent defects in T-cell and B-cell function and the excess production of cytokines, lead to a more prolonged pro-inflammatory responses and potentially poor outcomes [44].

Whether NLRP3 inflammasome over-activation is the cause of the hyper-inflammatory response to coronavirus infection in aged individuals, could be found in those animals, in the origin of the disease, bats.

Bats asymptomatically host a large number of high-risk, mainly RNA, viruses. These viruses are able to induce an aberrant innate immune over-activation which results in severe, and often deadly, disease in humans and animals [45].

When bats are infected by these viruses, they exhibit no or minimal signs of disease. Another interesting characteristic of bats is their extraordinarily long lifespan, relative to their body size, despite their elevated metabolic rates [46].

Bats display a significantly dampened activation of the NLRP3 inflammasome in primary immune cells, compared to human or mice [47]. This was also associated to a lower induction of ASC and inflammasome assembly, resulting in a decreased secretion of interleukin-1β in response to infection. This reduced inflammatory response, was seen after infections by several zoonotic RNA viruses including influenza A, PRV3M and Coronavirus (MERS). Remarkably, reduced inflammation as response to viral infection had no impact on the overall viral loads. So NLRP3/ASC/IL-1β dampened axis, is in the origin of the reduced inflammatory response to infection and the unexpected long lifespan in bats [47].

We could conclude that aged individuals have a basal predisposition to over-react to infection, displaying an increased chance of hyper-inflammatory syndromes, that seems not to be fully controlled. The NLRP3 inflammasome is over-activated in aged individuals, through deficient mitochondrial functioning, increased mROS and/or mtDNA. This mitochondrial failure leads to a hyper-responsive status in classically activated macrophages and subsequent increases in IL-1 β. This NLRP3 over-activated status in elderly individuals, is also observed in telomere dysfunctional mice models.

The ARDS related to coronavirus infection is the most common cause of death in severely ill patients. This syndrome is caused by exaggerated immune response due to an increased expression of pro-inflammatory cytokines dependent of activation of the NLRP3 inflammasome. Aged individuals show a constitutive increase of pro-inflammatory cytokines mediated by ROS/mtDNA induced NLRP3 activation. Coronavirus infection increased lethality observed in older age patients is due to more severe ARDS caused by an increased pro-inflammatory response mediated by the NLRP3 inflammasome. Strategies blocking inflammasome would deserve to be studied.
